# Determinants of Concurrent Motor and Language Recovery during Intensive Therapy in Chronic Stroke Patients: Four Single-Case Studies

**DOI:** 10.3389/fneur.2015.00215

**Published:** 2015-10-09

**Authors:** Annika Primaßin, Nina Scholtes, Stefan Heim, Walter Huber, Martina Neuschäfer, Ferdinand Binkofski, Cornelius J. Werner

**Affiliations:** ^1^Section Clinical-Cognitive Sciences, Department of Neurology, Uniklinik RWTH Aachen, Aachen, Germany; ^2^Department of Clinical Neurophysiology, University Medical Center, Göttingen, Germany; ^3^Department of Psychiatry, Psychotherapy and Psychosomatics, Medical Faculty, RWTH Aachen, Aachen, Germany; ^4^JARA – Translational Brain Medicine, Aachen, Germany; ^5^Department of Neurology, Medical Faculty, Uniklinik RWTH Aachen, Aachen, Germany; ^6^Institute of Neuroscience and Medicine (INM-1), Research Centre Jülich, Jülich, Germany; ^7^School for Physiotherapy, Uniklinik RWTH Aachen, Aachen, Germany; ^8^Institute of Neuroscience and Medicine (INM-4), Research Centre Jülich, Jülich, Germany

**Keywords:** stroke, motor, language, hemiplegia, hemiparesis, aphasia, recovery

## Abstract

Despite intensive research on mechanisms of recovery of function after stroke, surprisingly little is known about determinants of concurrent recovery of language and motor functions in single patients. The alternative hypotheses are that the two functions might either “fight for resources” or use the same mechanisms in the recovery process. Here, we present follow-up data of four exemplary patients with different base levels of motor and language abilities. We assessed functional scales and performed exact lesion analysis to examine the connection between lesion parameters and recovery potential in each domain. Results confirm that preservation of the corticospinal tracts (CSTs) is a neural predictor for good motor recovery while preservation of the arcuate fasciculus (AF) is important for a good language recovery. However, results further indicate that even patients with large lesions in CST, AF, and superior longitudinal fasciculus, respectively, are able to recover their motor/language abilities during intensive therapy. We further found some indicators of a facilitating interaction between motor and language recovery. Patients with positive improvement of motor skills after therapy also improved in language skills, while the patients with no motor improvements were not able to gain any language recovery.

## Introduction

It is a common clinical observation that in patients with both initial hemiparesis and aphasia after stroke, motor and language recovery may take different courses. Interestingly, scientific research has primarily focused on the examination of the course of recovery regarding either motor or language abilities, but only few studies addressed both. Aphasia has even been a criterion for exclusion in several studies of motor recovery (1, 2).

To our knowledge, there is only one multiple single-case study that addressed the issue of language recovery going parallel to a therapy of motor functions of the upper limb. Harnish et al. (3) examined five stroke patients during the course of 6 weeks of motor therapy. They assessed not only the recovery of motor functions of the upper limbs and functional motor reorganization but also changes in their language abilities. The authors report that in the three subjects showing the largest motor improvements they could also observe significant language improvements. In the individual fMRI measurements, where the patients had to tap the fingers of the paretic hand within the scope of their capacities, a shift of activation to the right hemisphere during the course of motor treatment could be observed in these three patients. Harnish et al. concluded that language changes seem to co-occur with motor changes after motor therapy. Anatomical analyses of the patients’ lesions were not carried out.

The finding of Harnish et al. that motor recovery can foster language recovery is very interesting for the current state of discussion about common mechanisms in motor and language processing. Especially the theory of cognitive embodiment has gained broad attention and kindled a whole line of research. In the light of embodiment theory, cognitive functions like language are grounded in the sensorimotor experiences and to the underlying systems (4, 5). For example, Hauk et al. (6) were able to show that language processing and comprehension activate motor regions, while Glenberg et al. (7) found that first- and second-grade children who manipulate images of toys on a computer screen develop improved comprehension skills in reading – a comprehension benefit was evoked by the conduction of motor tasks. There are numerous imaging studies demonstrating activation of the sensorimotor systems by listening to language with motor content [for example, see Ref. (8, 9)]. Recently anatomic correlates for common motor speech and motor (10) as well as language and motor processing (11) have been postulated on the basis of imaging data.

With the theoretical and experimental background that motor and language activity are not functionally independent, interdependencies regarding the course of recovery of these two domains can be assumed as well. These relations might result in two possible interactions between motor and language recovery processes: either competitive or additive effects may occur. Competitive rehabilitative interactions might be characterized by a “fight” for resources between the language and motor recovery capacities. In this case, a good motor recovery may limit or even prevent the course of language recovery and vice versa. The inverse assumption of an additive interaction between both domains during recovery implies that a positive course of motor recovery would influence language recovery positively, and vice versa. The results of Harnish et al. (3), which are in line with the findings concerning embodiment, seem to support the second hypothesis.

The identification of determinants of motor and language recovery after stroke is within the main stream of research on neurorehabilitation. There are several studies evaluating the role of lesion parameters as well as brain activation for complete or poor recovery for language and motor domain separately. We will briefly highlight the most relevant results in order to establish the backdrop for our study.

As to the motor domain, the lesion location is an important predictor for motor rehabilitation (12), whereas the size of the brain lesion seems to be no predictor for motor function recovery after stroke (12–14). Shelton and Reding (15) found that the probability of recovery of the upper limbs after stroke seems to diminish in dependence of the lesion location in the following order: cortex, corona radiata, and internal capsule. The dimension of impairment of the corticospinal tract (CST) is another indicator for good rehabilitation of hand motor function after stroke; severe damage of the CST has mainly been assessed in more severely affected patients (12–15).

Similarly, in the language domain, lesion location may play an important role in sufficient language recovery. Meinzer et al. (16) found that language rehabilitation after intensive language therapy was correlated with the integrity of the left hippocampus and the surrounding white matter. Marchina et al. (17) were able to show that the extent of impairment of the left arcuate fasciculus (AF) is a predictor for language recovery. The global lesion size does not have an influence on language rehabilitation after stroke [e.g., see Ref. (16, 18)].

Functional imaging has resulted in inconsistent results for both recovery of motor [e.g., see Ref. (19–23)] and language abilities [e.g., see Ref. (24–29)]. The heterogeneity of the results in the language and motor domain can possibly be attributed to different methods and objectives that were used in previous studies as well as different types of strokes (e.g., subcortical vs. cortical). Therefore, it is hardly possible to combine the mentioned results of the two different domains for predicting recovery patterns in patients with concurrent impairments in both domains.

Therefore, neural correlates for simultaneous recovery in the language and motor domain after stroke remain unclear. The results of Harnish et al. (3), which were investigated through fMRI and behavioral measurements, give a first hint for an additive interaction between both domains during the course of rehabilitation. To our knowledge, there are no studies with the aim to explore lesion characteristics of different ways of concurrent motor and language recovery. Therefore, it remains unclear if an additive interaction between motor and language recovery processes through therapy can be linked to specific structural lesions in the brain.

The aim of the present study was to investigate systematically the determinants of language and motor recovery in four exemplary patients with different base levels of motor and language abilities. Alongside the clinical assessment of motor and language abilities, we focused on (1) the examination of lesion characteristics at pre-test and (2) possible interactions of motor and language recovery processes following the 7-week language and motor therapy phase (i.e., outcome at the post-test).

Apraxia of speech is a clinically known influence factor to the possibilities of improving language skills in aphasic patients. Furthermore, since anatomic correlates for common motor speech and motor processing have been described (10), motor speech could be considered a “link” between motor and language processing functions. Therefore, in addition to motor and language processing functions, we considered the phenomenon of apraxia of speech independently for the patients in our patient group. Since we aimed to discuss motor speech functions on a purely exploratory level, no precise hypotheses were formulated.

Over all, four hypotheses were formulated concerning both lesion characteristics and possible therapy-induced interactions:

Regarding (1), lesion characteristics, the following hypotheses were addressed:

(i) In line with current research (12–14, 16, 18), we assume that global lesion size is not a correlate for sufficient concurrent motor and language recovery.
(ii) We expect that patients with smaller lesions in function-specific white matter tracts for motor (CST) and language processing [AF, superior longitudinal fasciculus (SLF)] show good recovery potential in the particular domains as opposed to patients with extensive lesions to these tracts.

Regarding (2), possible interactions of motor and language recovery processes, our hypotheses are as follows:

(iii) In line with Harnish et al. (3), we assume that patients with an increase in motor abilities after therapy phase will also show positive language recovery (i.e., an increase in language abilities at the post-test) and vice versa. This would indicate an additive interaction between motor and language domains during simultaneous motor and language therapy.
(iv) Complementarily, we anticipate that patients who do not profit from motor therapy do not show an increase in language abilities at the post-test after therapy phase and vice versa.

## Materials and Methods

### Patients

**Figure 1:**
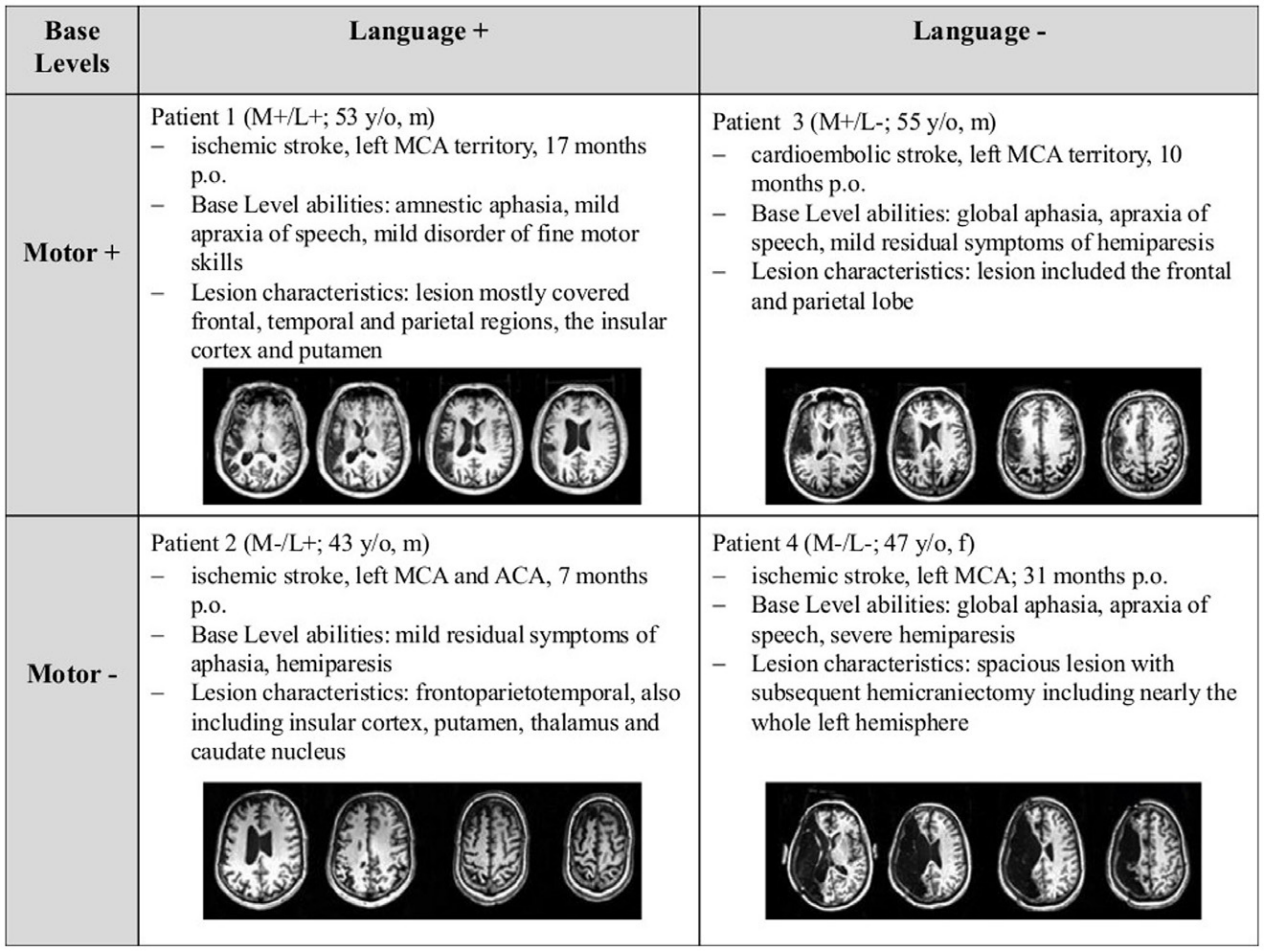
**Overview of the four patients’ base levels upon inclusion into the study, including T1-weighted images of the patients’ lesions, optimized for displaying the position of the lesion**. Abbreviations: “+” = good; “−” = poor motor/language skills; p.o., post onset; MCA, middle cerebral artery; ACA, anterior cerebral artery.

Four patients suffering from subacute to chronic stroke with different base levels of motor and language skills at the beginning of the study (see Figure 1) were selected. The selection of patients with opposing base levels in motor and language skills was conducted in order to include previous individual recovery processes into the evaluation of the current recovery process. Clinical records documented that at the acute stage of the stroke, all patients were described as non-fluent to globally aphasic and had paresis of varying degrees, ranging from mild hemiparesis (4/5) to full hemiplegia. The different base levels resulted from the patients’ individual recovery processes prior to the participation in the study.

At the beginning of the study, language skills were classified as “good” (Base: L+) or “poor” (Base: L−) according to the patients’ individual profile height in the language assessment of the Aachener Aphasie Test [AAT; (30) (see Table 1)]. Correspondingly, the classification of “good” vs. “poor” motor skills (M+ vs. M−) was based on the raw score of the Wolf Motor Function Test [WMFT (31), see Table 2]. This resulted in four possible baseline profiles: Base: M+/L+, M−/L+, M+/L−, and M−/L−, denoting good functions in both motor and language domains, the dissociations between the domains, and finally the combination of both severely impaired motor and language function at the pre-test of the study.

**Table 1:** **Results of the patients in the AAT and LEMO**.

**Pat. (base)**	**AAT**		**LEMO**							

	**Profile height**		**LD**		**FS**		**FR**		**ON**	
	**Pre**	**Post**	**Pre**	**Post**	**Pre**	**Post**	**Pre**	**Post**	**Pre**	**Post**
				
1 (M+/L+)	57.9	58.7	78	79	38	39	11	7°	19	20
2 (M−/L+)	72.5	73.3	80	80	40	40	20	20	18	18
3 (M+/L−)	41.9	43*	45	61*	34	36	10	6	–	–
4 (M−/L−)	40.9	41.3	70	74	35	37	–	–	9	9

AAT, Aachener Aphasie Test; LEMO, Lexikon Modellorientiert; Pat., patient; pre, pre-test; post, post-test; LD, Lexical Decision; FS, Finding Synonyms; FR, Finding Rhyms; ON, Oral Naming; *significant improvement [AAT: calculated with AATP; LEMO: McNemar Test, *p* < 0.05; (*)];°, significant deterioration (McNemar Test, *p* < 0.05).

**Table 2:** **Results of the patients in WMFT and DGI**.

**Pat. (base)**	**WMFT**		**DGI**	

	**Pre**	**Post**	**Pre**	**Post**
		
1 (M+/L+)	70	73	24	24
2 (M−/L+)	34	40*	20	24**
3 (M+/L−)	69	74**	21	23
4 (M−/L−)	5	5	11	13

Pat., patient; pre, pre-test; post, post-test; WMFT, Wolf Motor Function Test; DGI, Dynamic Gait Index; **significant improvement (Wilcoxon signed rank test, *p* < 0.05); *significant improvement (Wilcoxon signed rank test, *p* < 0.1).

Apart from different performance patterns in the language and motor domain, the patients had to meet the following criteria for inclusion into the study: (1) general MRI compatibility, (2) native German speakers, (3) right-handed according to the Edinburgh Inventory of Handedness [Laterality coefficient ≥80; (32)], (4) normal or corrected-to-normal vision, (5) no hearing loss, (6) no pregnancy, (7) single stroke in the left hemisphere, (8) subacute or chronic stage of stroke (at least 6 weeks post onset), (9) clinically diagnosed aphasia or residual symptoms of aphasia and clinically diagnosed hemiparesis, and (10) no history of dementia or other CNS or psychiatric diseases.

The patients were recruited from the Aphasia Rehabilitation Ward of the Neurological Clinic, Uniklinik RWTH Aachen. Informed written consent for participating in the study was obtained from each patient prior to the participation in the study. The study was approved by the local ethics committee and conducted according to the Declaration of Helsinki. Patient characteristics are displayed in Figure 1.

### Research design

**Figure 2:**
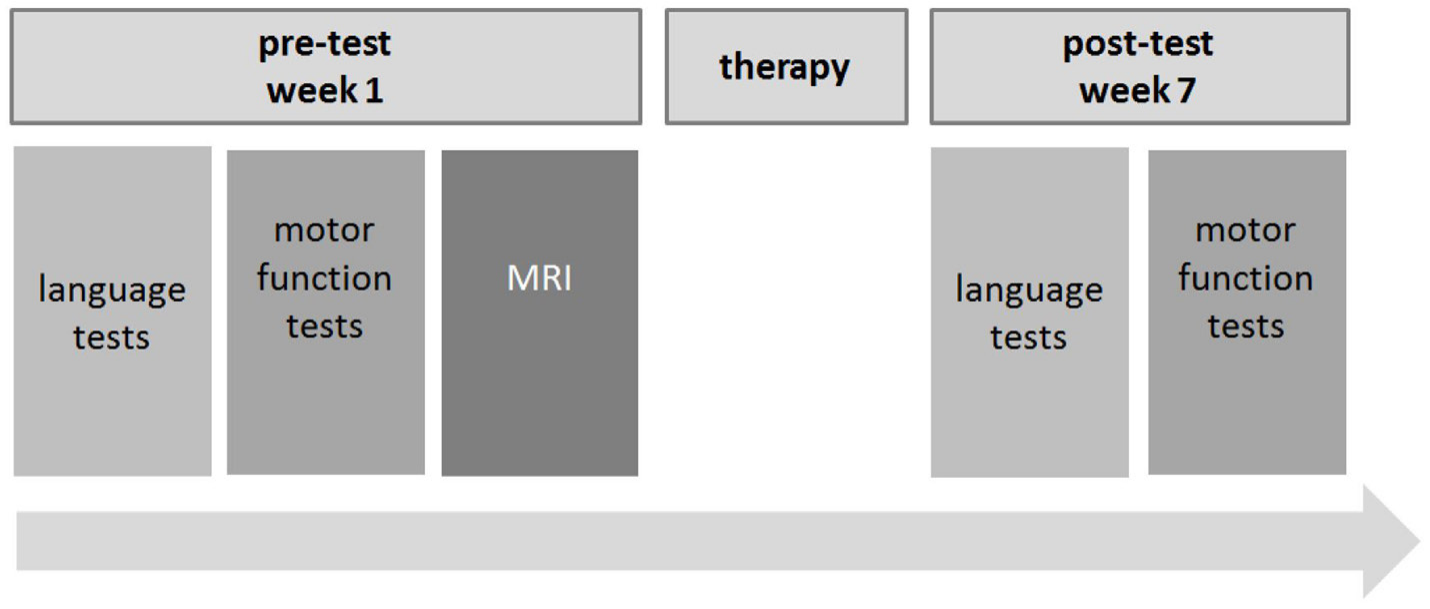
**Research design**.

All patients were recruited during their 7-week stay at the Aphasia Rehabilitation Ward of the Department of Neurology, Uniklinik RWTH Aachen. A pre–post test design was used to assess both motor and language abilities prior and after the 7-week therapy phase. The pre-test took place during the first week of the treatment. Deficits were quantified using standardized assessment tests and applied by trained personnel (speech and language therapists, physiotherapists, and neurologists). Structural MRI scans were conducted in the first week of the patients’ stay at the hospital. The post-test took place during the seventh (i.e., last) week of the stay at the Aphasia Rehabilitation Ward. Again, the functional language and motor scales were used to evaluate patients’ development during the intensive treatment. MRI measurements were not repeated. Between pre- and post-test, the patients participated in 7 weeks of motor and language therapy (for an overview of the research design, see Figure 2).

### Clinical examinations

The following tests were applied:

#### Functional Language Scales

The “Aachener Aphasie Test” [AAT (30)], a robust and highly validated test of language in multiple domains, was conducted to assess the patients’ overall linguistic abilities. Additionally, five subtests of the standard neurolinguistic test battery “Lexikon Modellorientiert” [LEMO (33)] were employed: subtest 5 – “lexical decision making,” subtest 25 – “finding synonyms,” subtest 30 – “oral naming,” and subtest 32 – “finding rhymes.”

#### Functional Motor Scales

The Wolf Motor Function Test [WMFT (31)] was applied to evaluate the quality and duration of the patients’ arm and hand movements. In addition, the Dynamic Gait Index [DGI (34)] was conducted in order to assess gait and balance.

#### Additional Scale

Three subtests from the “Aachener Materialien zur Diagnostik Neurogener Sprechstörungen” [AMDNS (35)] were used in order to screen for neurogenic speech disorders: “duration of phonation,” “variability of speech intensity,” and “articulatory diadochokinesis.” These subtests were used to control the influence of the patients’ motor speech function on motor and language ability and recovery.

In addition, subtest “Articulation” (spontaneous speech) of the AAT was considered separately, since it is specially related to motor speech functions.

#### Analysis of Behavioral Data

The single-case characteristics of our study put some restraints on the statistical tests that are available. For the AAT, significant improvements and deteriorations were differentiated. To test for significant changes in the patient’s performance between pre- and post-test, the computer program “AATP” (36) was employed. This program automatically calculates significant changes using the psychometric single-case diagnosis (37) with *p* < 0.1, an alpha-level that is common for single cases. In reference to LEMO, significant changes between pre- and post-test were calculated for each subtest conducting the McNemar test (*p* < 0.1 or *p* < 0.05). Concerning the WMFT and DGI, significant changes were calculated with the Wilcoxon signed rank test (*p* < 0.1 or *p* < 0.05). In the additional scale AMDNS, only notable changes were evaluated. They were defined as a positive or negative change of severity comparing the degrees of severity on a 4-point scale (3 = severe impairment, 0 = no impairment).

### Imaging acquisition

Structural MRI measurements (T1, FLAIR) were conducted for lesion analyses using a Philips 3T scanner at the Brain Imaging Facility at University Hospital, RWTH Aachen. All images were made using SENSE (Sensitivity Encoding) technology conducting an eight-channel phase array head coil. A three-dimensional isotropic T1-weighted sequence (MPRAGE) was performed in the sagittal plane. Acquisition parameters were: repetition time/echo time = 9.9/4.6 ms; flip angle = 8; field of view = 256 mm; matrix = 256 × 256; slice thickness = 1 mm; voxel size = 1 mm × 1 mm × 1 mm. Acquisition parameters for the FLAIR measurement were: repetition time/echo time = 11,000/125 ms; field of view = 224 mm; matrix = 312 × 157; slice thickness = 3 mm; voxel size = 0.72 mm × 1.13 mm × 3 mm.

### Analysis of imaging data

All data were analyzed on an individual subject basis. For the analysis of lesions, all lesions were marked within the FLAIR image using MRIcron (38). Afterwards, the lesion maps were normalized via FLIRT (39) and transformed into standard MNI space. Anatomical masks of interest from the atlases supplied with FSL [MNI Structural Atlas (40) and JHU White-Matter Tractography Atlas (41)] were extracted. The right hemisphere in the MNI Structural Atlas was masked out by zeroing all voxels with *x*-coordinates 0–45; anatomical structures of interest were already lateralized in the JHU White-Matter Tractography Atlas. No thresholding was applied. The size of each structure was determined by counting the number of non-zero voxels in each map. Then, an intersection of the patient-specific lesions (in standard space) with the respective anatomical maps was created by multiplying them with each other using FSL command line tools (fslmaths). This yielded a map representing the damage to the particular map inflicted by the patient’s lesion. The size of this map was determined by counting the non-zero voxels inside this map. Afterwards, the calculation of the percentage of the entire anatomical structure affected by the lesion followed by dividing the voxel count of the intersection by the voxel count of the anatomical map.

Lesions of the patients were analyzed according to their localization in the following cortical and subcortical structures: frontal lobe, parietal lobe, temporal lobe, occipital lobe, insula, putamen, thalamus, and caudate. Concerning white matter tracts, the lesion analysis procedure previously described was conducted for the CSTs, SLF and AF. All fiber tracts were included due to their previously described role in motor and language processing.

## Results

### Behavioral data

The patients’ overall behavioral outcome (changes of performance after the 7-week therapy phase) in the functional scales showed heterogeneous results both for motor and language assessments (see Tables 1 and 2; Tables S1–S3 in Supplementary Material).

### Additional scale

Motor speech abilities (AMDNS) showed heterogeneous results with both notable improvements and deteriorations across all patients’ performances. However, none of the measured changes occurred on a significant level. An overview of the results in these tests is given in Table 3. As described above, subtest “Articulation” of the AAT was considered separately and showed heterogeneous results with notable improvements in Patient 1 (Base: M+/L+) and Patient 3 (Base: M+/L−), one notable deterioration [Patient 2 (Base: M−/L+)] and one stable result [Patient 4 (Base: M−/L−; see Table 4)].

**Table 3:** **Results of the patients in the AMDNS (degree of impairment)**.

**Pat. (base)**	**AMDNS**					

	**DIA**		**DU**		**INT**	
	**Pre**	**Post**	**Pre**	**Post**	**Pre**	**Post**

1 (M+/L+)	18	15	9	5	0	3
2 (M−/L+)	18	18	3	3	3	0
3 (M+/L−)	6	6	6	6	0	2
4 (M−/L−)	9	9	16	17	3	3

*Cumulative dysarthria score: degree of impairment: 0 = no impairment; 3 = severe impairment. Pat., patient; pre, pre-test; post, post-test; DIA, diadochokinesis; DU, duration of phonation; INT, variability of speech intensity*.

**Table 4:** **Results of the patients in the subtest “Articulation” (AAT; degree of impairment)**.

**Pat. (base)**	**Articulation (AAT)**	
	**Pre**	**Post**

1 (M+/L+)	3	4
2 (M−/L+)	5	4
3 (M+/L−)	2	3
4 (M−/L−)	3	3

*Degree of impairment: 5 = no impairment; 1 = severe impairment (i.e., cannot be evaluated due to lack of intelligibility)*.

### Synoptical analysis of behavioral and lesion-related data

**Figure 3:**
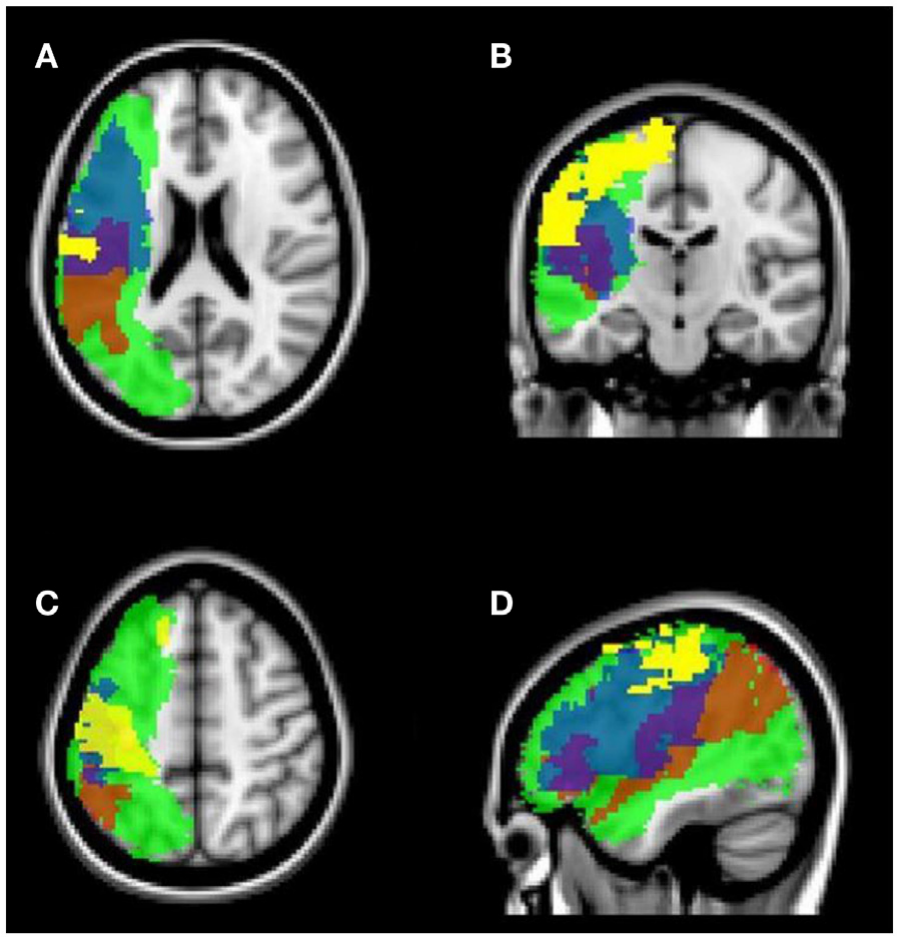
**Structural MRI (FLAIR sequence)**. Overlay of normalized lesion maps of the patients in the standard brain. Red, Patient 1 (Base: M+/L+); yellow, Patient 2 (Base: M−/L+); blue, Patient 3 (Base: M+/L−); green, Patient 4 (Base: M−/L−). **(A)** axial, subcortical view; **(B)** axial, cortical view; **(C)** coronal view; **(D)** sagittal view (left hemisphere).

**Figure 4:**
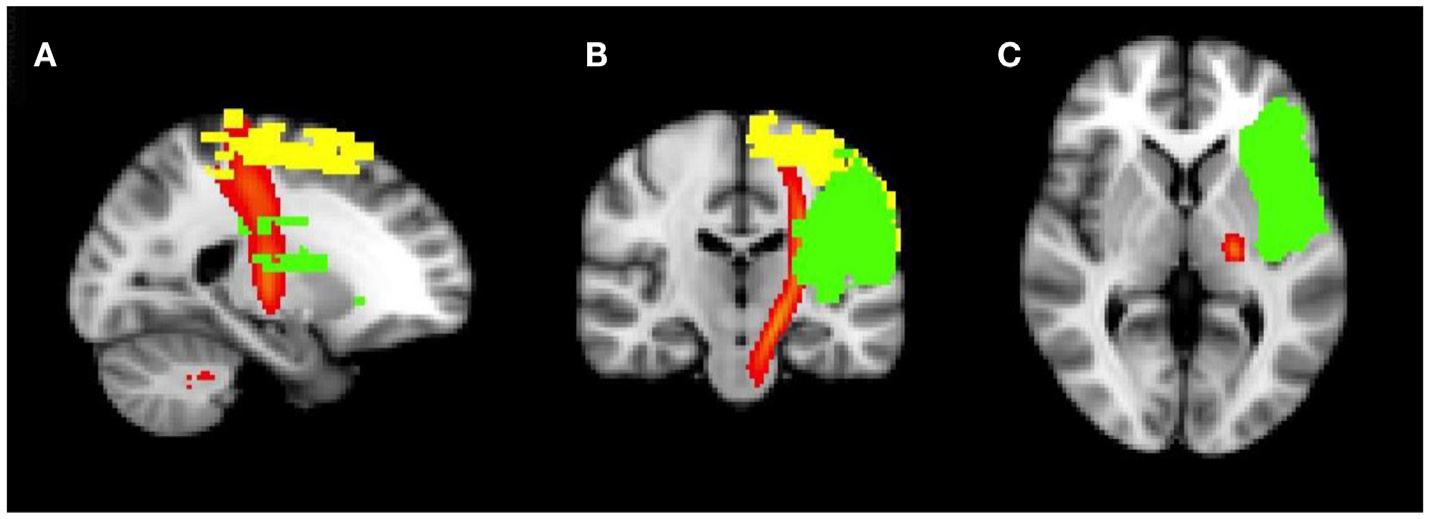
**Structural MRI (FLAIR sequence)**. Overlay of normalized lesion maps of patients 2 and 3 in the standard brain. Yellow, Patient 2 (Base: M−/L+); green, Patient 3 (Base: M+/L−); red, corticospinal tract. **(A)** sagittal view; **(B)** coronal view; **(C)** axial, subcortical view.

As shown in Tables 5 and 6, all patients had lesions in the frontal and parietal lobe, as well as white matter tract injury in the SLF and AF (see also Figures 3 and 4). Concerning further cortical and subcortical structures, patients did not show a homogeneous pattern of their lesions. In the following tables, we demonstrate an overview of the patients’ recovery outcome following the 7-week therapy phase together with the patients’ lesion characteristics in cortical and subcortical (Table 5) as well as white matter tract areas (Table 6).

**Table 5:** **Overview of the patients’ functional recovery (post-test) in both domains, lesion volume, and percentage of damaged tissue in defined cortical and subcortical brain areas**.

**Pat. (base)**				**Lesion volume to specific areas**							
	**Outcome after 7-week therapy phase**		**Total lesion volume**	**Cortical (Lobar)**				**Subcortical**			
	**Motor recovery**	**Language recovery**		**Fro**	**Par**	**Tem**	**Occ**	**Ins**	**Put**	**Tha**	**Cau**

1 (M+/L+)	Non-responder WMFT (o), DGI (o)	Non-responder AAT (o), LEMO−	10,325	2,403	6,368	2,409	335	1,903	437	–	–
2 (M−/L+)	Strong responder WMFT+, DGI+	Non-responder AAT (o), LEMO (o)	6,852	5,815	3,193	–	–	–	–	–	–
3 (M+/L−)	Partial responder WMFT+, DGI (o)	Strong Responder AAT+, LEMO+	14,406	8,422	4,171	1,255	–	2,764	732	23	25
4 (M−/L−)	Non-responder WMFT (o), DGI (o)	Non-responder AAT (o), LEMO (o)	50,472	18,747	16,884	9,692	7,556	3,340	1,237	87	12

*+, Significant improvement; (o), no change; −, significant deterioration. Non-responder, patient showed no positive response to motor or language therapy; partial responder, partial positive response, i.e., significant improvement in one of the applied tests; strong responder, strong positive response, i.e., significant improvement in both applied tests; –, no lesion measured; Fro, frontal lobe; Par, parietal lobe; Tem, temporal lobe; Occ, occipital lobe; Ins, insula; Put, putamen; Tha, thalamus; Cau, nucleus caudate*.

*Lesion volume was calculated within the FLAIR data (voxels)*.

**Table 6:** **Overview of the patients’ functional recovery (post-test) in both domains, lesion volume, and percentage of damaged tissue in particular white matter tracts**.

Pat. (base)	Outcome after 7-week therapy phase		Lesion volume to specific white matter tracts		
	Motor recovery	Language recovery	CST	SLF	AF
1 (M+/L+)	Non-responder WMFT (o), DGI (o)	Non-responder AAT (o), LEMO−	–	5,115	1,196
2 (M−/L+)	Strong responder WMFT+, DGI+	Non-responder AAT (o), LEMO (o)	1,057	1,916	736
3 (M+/L−)	Partial responder WMFT+, DGI (o)	Strong responder AAT+, LEMO+	568	7,188	3,944
4 (M−/L−)	Non-responder WMFT (o), DGI (o)	Non-responder AAT (o), LEMO (o)	1,643	16,462	7,458

*+, Significant improvement; (o), no change; −, significant deterioration. Non-responder, patient showed no positive response to motor or language therapy; partial responder, partial positive response, i.e., significant improvement in one of the applied tests; strong responder, strong positive response, i.e., significant improvement in both applied tests; –, no lesion measured; CST, corticospinal tract; SLF, superior longitudinal fasciculus; AF, arcuate fasciculus*.

*Lesion volume was calculated within the FLAIR data (voxels)*.

## Discussion

The present study explored if there are determinants for concurrent motor and language recovery during intensive therapy in four exemplary chronic stroke patients with different base levels of language and motor abilities. In particular, we examined if (1) concerning lesion characteristics (i) the global lesion size is a correlate of sufficient concurrent motor and language recovery and if (ii) the extent of damage of the function-specific white matter tracts for motor and language is predictive for the recovery potential in the respective domains.

In the further analysis of (2) possible interactions of motor and language recovery processes, we investigated if (iii) an additive interaction between motor and language domains during simultaneous motor and language therapy occurs and if (iv) there will be a lack of interaction between both domains when there is no recovery progress in at least one domain.

The four patients had different motor and language base levels and were systematically examined in this study to evaluate the relation of their therapy outcome in both domains (i.e., recovery process that was measured from pre- to post-test) and lesion parameters. To explore predictors for (iii) concurrent motor and language recovery, various functional scales in the motor and language domain and also in the motor speech domain were applied. Concerning the lesion analysis, cortical and subcortical lesion characteristics as well as white matter tract damage were explored.

One major finding of this study is that we could detect some indicators for an additive behavior of motor and language recovery. It seems that motor and language recovery co-occur in a sense that motor recovery facilitates the possibility of a positive therapy-induced language recovery. In addition, lesion size *per se* is not determining a sufficient motor and language recovery. However, the specific lesion areas play an important role for a sufficient recovery. Another main finding was that large damage in important fiber structures for motor or language processing allows no prediction about the recovery of the fiber-induced function at a single subject level.

### Lesion characteristics

#### Global Lesion Size

Considering the global lesion size in our four patients, Patient 3 (Base: M+/L−) was the only participant who was able to improve significantly in both motor and language functions at the post-test. In addition, this patient had the second largest overall lesion size. In comparison, Patient 2 (Base: M−/L+), the patient showing the smallest global lesion size, was able to improve in motor but not language scales at the post-test, whereas Patient 1 (Base: M+/L+) and Patient 4 (Base: M−/L−, the patient with the largest global lesion size), did not show improvement in any scale. The fact that Patient 3 (Base: M+/L−) was able to improve on such an extensive level shows that the global lesion size cannot be the single determinant regarding recovery potential. This finding is in line with the current state of research [e.g., see Ref. (12–14, 16, 18)].

#### White Matter Tracts

Concerning white matter tracts, lesion characteristics seem to be less distinct. Although Patient 1 (Base: M+/L+) was the only patient who did not show a lesion of the CST, he also did not improve in motor therapy, most possibly due to a high motor base level and a ceiling effect. Patient 3 (Base: M+/L−) showed the smallest lesion of all patients (i.e., of all patients with lesions of the CST) and was able to improve in one motor test. Whereas Patient 2 (Base: M−/L+) with the second largest lesion of the CST was a strong responder to motor therapy with improvements in both motor function tests. Patient 4 (Base: M−/L−) had the most extensive CST lesion and was a non-responder to motor therapy.

Especially the distinction between Patients 2 (Base: M−/L+) and 3 (Base: M+/L−) is of further interest: although fiber damage of the CST in Patient 2 (Base: M−/L+) was about two times larger than that in Patient 3 (Base: M+/L−), probably leading to his worse baseline profile, Patient 2 (Base: M−/L+) actually showed better abilities to recover in the motor domain than Patient 3 (Base: M+/L−; strong responder vs. partial responder, see Tables 5 and 6). This difference could be attributed to the fact that the measureable extent of the lesion in Patient 2 is primarily caused by the location of the lesion at the level of the primary motor cortex, whereas Patient 3’s smaller lesion mainly affects the part of the pyramidal tract further down in the corona radiata (see Figure 4). It is possible that this specificity of the anatomical lesion site in Patient 3 leads to a higher amount of damage to fibers that are relevant to motor recovery.

In summary, among our patient group, Patient 1 (Base: M+/L+) showed no lesion of the CST and no therapy-induced improvement due to ceiling effects and an already high level of motor functions at the pre-test. Patient 2 (Base: M+/L−) showed an extensive overall lesion, however, damage was more related to cortical structures than to lesions in the CST. This patient showed good recovery potential with improvements in both motor function tests. In comparison, Patient 3 (Base: M+/L−) showed a smaller lesion, however, he only recovered to a smaller degree than Patient 2 (Base: M−/L+). His lesion location in the corona radiata probably led to a reduction in recovery potential. Last, Patient 4 (Base: M−/L−) with the most extensive lesion of the CST was not able to improve in motor therapy at all. This result is supportive to the finding that strategic lesion location, rather than lesion volume, is an important determinant to recovery potential [e.g., see Ref. (15)].

Concerning the lesion of the AF, similar results could be found. Patient 2 (Base: M−/L+) who showed the smallest lesion of the AF did not show therapy-induced language improvement at the post-test, as well as Patient 1 (Base: M+/L+) who presented with the second smallest lesion. Patient 4 (Base: M−/L−) who showed the most spacious lesion of the AF was also not able to profit significantly from intensive therapy. Only Patient 3 (Base: M+/L−) was able to improve strongly in both language scales, although he showed the second largest lesion of the AF.

These findings seemingly point toward the assumption that the specific lesion size of the CST and/or AF does not directly influence the outcome of motor and/or language recovery. However, we feel that this assumption would be too shortsighted since at this point, the individual base levels, i.e., the level of motor and language skills that the patients presented with at the pre-test, need to be considered: regarding the lesion of the CST, we pointed out that Patient 1 (Base: M+/L+) did not show motor recovery although he did not have any lesion of the CST. However, Patient 1 already showed a comparatively high level of motor skills at the pre-test (see Table 2), leaving him with only small possibilities for significant improvements at the post-test. The same holds for Patient 2 (Base: M−/L+) regarding the extent of the AF lesion. As described, Patient 2 showed the smallest AF lesion of all patients but did not show language recovery. This could be attributed to possible ceiling effects. However, even after eliminating those two patients with possible ceiling effects from our considerations, in our patient group still neither the patient with the (then) smallest CST lesion [Patient 3 (Base: M+/L−)] nor the patient with the (then) smallest AF lesion [Patient 1 (M+/L+)] are the patients showing most motor and language recovery, respectively. This observation points strongly toward the conclusion, that even patients with large lesion of the CST/AF are able to recover motor/language abilities during intensive therapy.

### Interactions of motor and language recovery processes

Based on the results that were published by Harnish et al. (3), we assumed that the patients with an increase in motor abilities after the 7-week therapy phase would show positive language recovery (i.e., an increase in language abilities at the post-test), indicating that an additive interaction between motor and language domains during simultaneous motor and language therapy occurs. We also anticipated that patients who do not profit from motor therapy do not show an increase in language abilities at the post-test after therapy phase and vice versa. Regarding the data of our four patients, two of our patients, namely Patient 2 (Base: M−/L+) and Patient 3 (Base: M+/L−), were able to profit from motor therapy, leading to a significant improvement of motor functions at the post-test. Of these two patients, Patient 2 (Base: M−/L+) did not show improvements in the language domain while Patient 3 (Base: M+/L−) was a strong responder to language therapy also (see Tables 5 and 6). However, Patient 2 (Base: M−/L+) already showed a comparatively high level of language skills at the pre-test with a mean profile height of 72.5 in the AAT (see Table 1) as well as even the maximum possible raw scores at LeMo, indicating only mild residual symptoms of aphasia even at the beginning of the therapy phase.

As to Patient 1 (Base: M+/L+) and Patient 4 (Base: M−/L−), none of them were able to improve motor function skills and, in addition, none of them were able to profit from language therapy. Of the two patients, Patient 1 (Base: M+/L+) already showed a relatively high language profile at the pre-test, however, with a mean profile height of 57.9 in the AAT, he clearly could have improved significantly in that scale. Additionally, the raw scores indicate that significant improvement of the subtest “Finding Rhymes” (LeMo) would also have been possible (see Table 1). Therefore, the existence of ceiling effects in this patient can be excluded and the lack of positive therapy outcome has to be considered as a “real” effect.

In none of our four patients improvements in the motor or the language domains were bound to measurable deteriorations in the other domain. This lack of dissociation between the recovery processes of the two domains hints toward the assumption that a “fight for resources” could not be observed in our patient group.

In conclusion, only one patient with a positive response to motor therapy [Patient 3 (Base: M+/L−)] was able to improve significantly in language functions at the pre-test, whereas Patient 2 (Base: M−/L+), who also improved significantly in motor functions, could not have achieved measurable improvements due to ceiling effects in the language domain but did show numerical improvements of language skills. The evident motor recovery in the case of Patient 3 (Base: M+/L−) might have been a facilitating factor for a good response to language therapy. The two patients who could not benefit from the intensive motor therapy program [Patient 1 (Base: M+/L+) and Patient 4 (Base: M−/L−)] could also not improve significantly concerning language skills. Therefore, we assume that these results are suggestive of a positive interaction operating between motor and language domains during recovery in the sense that a positive therapy-induced motor recovery is a prerequisite to the possibility of recovering language skills through language therapy. This finding is in accordance with Harnish et al. (3). Regarding the oppositional outcome (positive language therapy outcome leading to improved motor outcome), no such interactions could be observed, therefore, due to our small sample size, it is not possible to formulate a conclusion concerning the possibility of contrary recovery dynamics.

### Apraxia of speech

Interestingly, dissociations in the recovery of apraxia of speech became apparent in the additional functional Scale AMDNS and in the subtest “articulation” of the AAT.

Only Patient 1 (Base: M+/L+), who had the smallest amount of lesioned voxels in the frontal lobe (see Table 5), was able to improve notably in “articulatory diadochokinesis” and “duration of phonation” (AMDNS) and showed a notable improvement in the communication parameter “articulation” (AAT; see Tables 3 and 4). Patient 2 (Base: M−/L+), showing a larger lesion in the frontal lobe, showed stable performances regarding motor speech. Patient 3 (Base: M+/L−) and 4 (Base: M−/L−) had the highest amount of lesioned voxels in the frontal lobe and stable or inferior results in the post-test [except of a notable improvement in the communication parameter “articulation” (AAT, Patient 3)]. Patients 3 (Base: M+/L−) and 4 (Base: M−/L−) were also not able to conduct complex articulatory diadochokinesis tasks at the pre- and post-test, probably due to the severe apraxia of speech. These two patients demonstrate larger affection of the insular cortex by the lesion in comparison to Patient 2 (Base: M−/L+; no insular lesion) and Patient 1 (Base: M+/L+; see Table 5). The insula is associated with articulatory coding/motor programing and motor control [e.g., see Ref. (42, 43)] and its left precentral gyrus forms also an anatomical correlate for the development of apraxia of speech (44, 45). Therefore, preservation of the insula appears to be a necessary, but not exclusive predictor for motor speech recovery. Lesions in other cortical or subcortical regions may also play a role for developing recovery potential in motor speech coordination. This assumption would be in accordance with the findings of Ogar et al. (45). They pointed out that patients showing a severe apraxia of speech had larger lesions in neighboring regions like Broca’s area or basal ganglia. To conclude, the described literature and our findings suggest that the overall amount of lesioned voxels in the frontal lobe *per se* is able to predict motor speech recovery in our sample of patients. This finding has to be tested in a larger number of patients and, in addition, distinctive subcortical parts of the frontal lobe like insular or basal ganglia should be analyzed precisely in reference to their predictive value for recovery.

### Limitations

The present multiple case study provides a new approach in analyzing concurrent motor and language recovery as well as the interaction behavior between these domains during recovery. On the one hand, our findings provide some first indictors, given the fundamental research gap in this field. On the other hand, the data in this study are of limited generalizability as only single cases were examined. In addition, a more specific analysis of specific brain areas is needed. It was also not possible to control the time of onset/duration of aphasia and motor dysfunction in the patients. This is a variable of potential influence due to different restitution processes in different time intervals after stroke [e.g., restitution in the early subacute vs. chronic stage of aphasia; see Ref. (46)]. A group study would be necessary to elucidate if these first results are transferable to a larger sample of subjects.

## Conclusion and Perspectives

To conclude, we show that primarily the strategic location of the lesion is a determinant of functional recovery in the motor and language domain. Another main finding was that large damage to important white matter structures for motor or language processing is not a single predictive factor for the recovery of the affected function. Regarding motor speech, the extent of damage to the frontal lobe (especially insula) seems to be a neural correlate for a good motor speech (apraxia of speech) recovery. Poor motor speech abilities, often associated with an apraxia of speech, play a special role in the recovery of language skills and are distinguished by large frontal lesions.

With respect to the interaction of the motor and language domain during recovery, first hints for additive effects were found. Those patients with good base levels in motor skills improved in language abilities. Therefore, motor and language improvement seem to co-occur, as stated before by Harnish and colleagues (3), rather than to compete for recovery resources.

Concerning the mechanisms of recovery, we were not able to find evidence for a “fight for resources,” since motor or language recovery was not associated with a loss of abilities in the other domain, respectively. But it was clearly visible that there is no prospect of recovery in the language domain if there are no resources and abilities available in the motor domain. This is indicative for an additive, synergetic recovery mechanism as described by Harnish and colleagues (3).

A further important finding was that the characteristics of the lesion (specific area, overall size) are no obligatory determinant or predictor for the success of motor or language therapy. We could show that a patient with large CST damage exhibited positive motor recovery while a patient with large AF/SLF damage improved well in the language testing.

In this study, only single cases were analyzed. A larger group study will investigate recovery mechanisms and correlates supported by a higher statistical power as well as additional fMRI measurements. The results, together with the findings in this paper, will add to the knowledge about recovery processes in this clinically relevant patient group.

## Conflict of Interest Statement

The authors declare that the research was conducted in the absence of any commercial or financial relationships that could be construed as a potential conflict of interest.

## Supplementary Material

The Supplementary Material for this article can be found online at http://journal.frontiersin.org/article/10.3389/fneur.2015.00215

## References

[1] Calautti C Jones PS Naccarato M Sharma N Day DJ Bullmore ET The relationship between motor deficit and primary motor cortex hemispheric activation balance after stroke: longitudinal fMRI study. J Neurol Neurosurg Psychiatr (2007) 81(7):788–92. 10.1136/jnnp.2009.190512 20392975

[2] Ward NS Brown MM Thompson AJ Frackowiak RS. Neural correlates of outcome after stroke: a cross-sectional fMRI study. Brain (2003) 126(Pt 6):1430–48. 10.1093/brain/awg145 12764063 PMC3717456

[3] Harnish S Meinzer M Trinastic J Fitzgerald D Page S. Language changes coincide with motor and fMRI changes following upper extremity motor therapy for hemiparesis: a brief report. Brain Imaging Behav (2011) 8(3):370–7. 10.1007/s11682-011-9139-y 21989635

[4] Borghi AM Riggio L. Sentence comprehension and simulation of object temporary, canonical and stable affordances. Brain Res (2009) 1253:117–28. 10.1016/j.brainres.2008.11.064 19073156

[5] Bersalou LW Perceptual symbol systems. Behav Brain Sci (1999) 22:577–609. 11301525 10.1017/s0140525x99002149

[6] Hauk O Johnsrude I Pulvermüller F. Somatotopic representation of action words in human motor and premotor cortex. Neuron (2004) 41(2):301–7. 10.1016/S0896-6273(03)00838-9 14741110

[7] Glenberg AM Goldberg AB Zhu X Improving early reading comprehension using embodied CAI. Instr Sci (2011) 39:27–39. 10.1007/s11251-009-9096-7

[8] Jirak D Menz MM Buccino G Borghi AM Binfkofski F. Grasping language – a short story on embodiment. Conscious Cogn (2010) 19:711–20. 10.1016/j.concog.2010.06.020 20739194

[9] Tettamanti M Buccino G Saccuman MC Gallese V Danna M Scifo P Listening to action-related sentences activates fronto-parietal motor circuits. J Cogn Neurosci (2005) 17(2):273–81. 10.1162/0898929053124965 15811239

[10] Heim S Amunts K Hensel T Grande M Huber W Binkofski F The role of human parietal area 7A as a link between sequencing in hand actions and in overt speech production. Front Psychol (2012) 3:534. 10.3389/fpsyg.2012.00534 23227016 PMC3514541

[11] Hoeren M Kümmerer D Bormann T Beume L Ludwig VM Vry MS Neural bases of imitation and pantomime in acute stroke patients: distinct streams for praxis. Brain (2014) 137(t10):2796–810. 10.1093/brain/awu203 25062694

[12] Binkofski F Seitz RJ Hackländer T Pawelec D Mau J Freund HJ. Recovery of motor functions following hemiparetic stroke: a clinical and magnetic resonance-morphometric study. Cerebrovasc Dis (2001) 11(3):273–81. 10.1159/000047650 11306779

[13] Binkofski F Seitz RJ Arnold S Classen J Benecke R Freund HJ. Thalamic metbolism and corticospinal tract integrity determine motor recovery in stroke. Ann Neurol (1996) 39(4):460–70. 10.1002/ana.410390408 8619524

[14] Zhu LL Lindenberg R Alexander MP Schlaug G. Lesion load of the corticospinal tract predicts motor impairment in chronic stroke. Stroke (2010) 41(5):910–5. 10.1161/STROKEAHA.109.577023 20378864 PMC2886713

[15] Shelton FN Reding MJ. Effect of lesion location on upper limb motor recovery after stroke. Stroke (2001) 32(1):107–12. 10.1161/01.STR.32.1.107 11136923

[16] Meinzer M Mohammadi S Kugel H Schiffbauer H Flöel A Albers J Integrity of the hippocampus and surrounding white matter is correlated with language training success in aphasia. Neuroimage (2010) 53(1):283–90. 10.1016/j.neuroimage.2010.06.004 20541018

[17] Marchina S Zhu LL Norton A Zipse L Wan CY Schlaug G. Impairment of speech production predicted by lesion load of the left arcuate fasciculus. Stroke (2011) 42(8):2251–6. 10.1161/STROKEAHA.110.606103 21719773 PMC3167233

[18] Lazar RM Speizer AE Festa JR Krakauer JW Marshall RS. Variability in language recovery after first-time stroke. J Neurol Neurosurg Psychiatry (2008) 79(5):530–4. 10.1136/jnnp.2007.122457 17846113

[19] Rehme AK Fink GR Cramon DY von Grefkes C. The role of the contralesional motor cortex for motor recovery in the early days after stroke assessed with longitudinal fMRI. Cereb Cortex (2011) 21(4):756–68. 10.1093/cercor/bhq140 20801897

[20] Schaechter JD Perdue KL Wang R. Structural damage to the corticospinal tract correlates with bilateral sensorimotor cortex reorganization in stroke patients. Neuroimage (2008) 39(3):1370–82. 10.1016/j.neuroimage.2007.09.071 18024157 PMC2259387

[21] Gerloff C Khalaf B Sailer A Wassermann EM Chen R Matsuoka T Multimodal imaging of brain reorganization in motor areas of the contralesional hemisphere of well recovered patients after capsular stroke. Brain (2006) 129(3):791–808. 10.1093/brain/awh713 16364955

[22] Grefkes C Ward NS Cortical reorganization after stroke: how much and how functional? Neuroscientist (2013) 20(1):56–70. 10.1177/1073858413491147 23774218

[23] Stoeckel MC Binkofski F. The role of ipsilateral primary motor cortex in movement control and recovery from brain damage. Exp Neurol (2010) 221(1):13–7. 10.1016/j.expneurol.2009.10.021 19896482

[24] Rosen HJ Petersen SE Linenweber MR Snyder AZ White DA Chapman L Neural correlates of recovery from aphasia after damage to left inferior frontal cortex. Neurology (2000) 55(12):1883–94. 10.1212/WNL.55.12.1883 11134389

[25] Saur D Ronneberger O Kümmerer D Mader I Weiller C Klöppel S. Early functional magnetic resonance imaging activations predict language outcome after stroke. Brain (2010) 133(4):1252–64. 10.1093/brain/awq021 20299389

[26] Zipse L Norton A Marchina S Schlaug G. When right is all that is left: plasticity of right-hemisphere tracts in a young aphasic patient. Ann N Y Acad Sci (2012) 1252:237–45. 10.1111/j.1749-6632.2012.06454.x 22524365 PMC3589758

[27] Raboyeau G Boissezon X de Marie N Balduyck S Puel M Bezy C Right hemisphere activation in recovery from aphasia: lesion effect or function recruitment? Neurology (2008) 70(4):290–8. 10.1212/01.wnl.0000287115.85956.87 18209203

[28] Saur D Hartwigsen G. Neurobiology of language recovery after stroke: lessons from neuroimaging studies. Arch Phys Med Rehabil (2012) 93(1 Suppl):S15–25. 10.1016/j.apmr.2011.03.036 22202187

[29] Brownsett SLE Warren JE Geranmayeh F Woodhead Z Leech R Wise RJS. Cognitive control and its impact on recovery from aphasic stroke. Brain (2014) 137(Pt 1):242–54. 10.1093/brain/awt289 24163248 PMC3891442

[30] Huber W Poeck K Weniger D Willmes K Aachener Aphasie Test. Göttingen: Hogrefe (1983).

[31] Wolf SL Catlin PA Ellis M Archer AL Morgan B Piacentino A. Assessing wolf motor function test as outcome measure for research in patients after stroke. Stroke (2001) 32(7):1635–9. 10.1161/01.STR.32.7.1635 11441212

[32] Oldfield RC The assessment and analysis of handedness: the Edinburgh inventory. Neuropsychologia (1971) 9(1):97–113. 10.1016/0028-3932(71)90067-4 5146491

[33] Bleser R de Cholewa J Stadie N Tabatabaie S LEMO: Lexikon Modellorientiert. München: Elsevier (2010).

[34] Shumway-Cook A Woollacott MH Motor Control: Theory and Practical Applications. Philadelphia, PA: Lippincott Williams & Wilkins (2001). 614 p.

[35] Schnitker R Huber W Pustelniak M Weyer D Willmes K Bülte D Die aachener materialien zur diagnostik neurogener sprechstörungen (AMDNS). Neurol Rehabil (2011) 5/6:277.

[36] Guillot G Willmes K Kremer C AATP 4.0 Für Windows [Computer Program]. Bonn: Phoenix Software GmbH (2008).

[37] Huber HP Psychometrische Einzelfalldiagnostik. Weinheim: Beltz (1973).

[38] Rordon C MRIcron [Computer Program]. Version 7. (2012). Available from: http://www.mricro.com

[39] Jenkinson M Smith SM. A global optimisation method for robust affine registration of brain images. Med Image Anal (2001) 5(2):143–56. 10.1016/S1361-8415(01)00036-6 11516708

[40] Mazziotta JC Toga AW Evans A Fox P Lancaster J A probabilistic atlas of the human brain: theory and rationale for its development. The international consortium for brain mapping (ICBM). Neuroimage (1995) 2(2):89–101. 10.1006/nimg.1995.1012 9343592

[41] Oishi K Faria A van Zijl PC Mori S MRI Atlas of Human White Matter. London: Elsevier (2011). 257 p.

[42] Petersen SE. Positron emission tomographic studies of the processing of singe words. J Cogn Neurosci (1989) 1(2):153. 10.1162/jocn.1989.1.2.153 23968463

[43] Ackermann H Riecker A. The contribution of the insula to motor aspects of speech production: a review and a hypothesis. Brain Lang (2004) 89(2):320–8. 10.1016/S0093-934X(03)00347-X 15068914

[44] Dronkers N. A new brain region for coordinating speech articulation. Nature (1996) 384(6605):159–61. 10.1038/384159a0 8906789

[45] Ogar J Willock S Baldo J Wilkins D Ludy C Dronkers N. Clinical and anatomical correlates of apraxia of speech. Brain Lang (2006) 97(3):343–50. 10.1016/j.bandl.2006.01.008 16516956

[46] Cherney LR Small SL Aphasia, apraxia of speech and dysarthria. In: Harvey RL Macko RF Stein J Zorowitz RD Winstein CJ, editors. Stroke Recovery and Rehabilitation. New York, NY: Demos Medical Publishing (2008). p. 155–82.

