# Supplementary material for: Determinants of Concurrent Motor and Language Recovery during Intensive Therapy in Chronic Stroke Patients: Four Single-Case Studies
Source: Front Neurol. 2015 Oct 9;6:215. doi: 10.3389/fneur.2015.00215 (PMC4598579; doi:10.3389/fneur.2015.00215)
Supplement: Supplementary file 1 [file Data_Sheet_1.DOCX]

***Supplementary Material***

**Neural correlates of motor and language recovery after stroke: four single case studies**

**Annika Primaßin^17#^, Nina Scholtes^1#^, Stefan Heim^134^, Walter Huber^2^, Martina Neuschäfer^6,^ Ferdinand Binkofski^15#^* & Cornelius J. Werner^2#^**

^1^Section Clinical-Cognitive Sciences, Department of Neurology, Uniklinik RWTH Aachen, Germany

^2^Aphasia Rehabilitation Ward, Department of Neurology, Uniklinik RWTH Aachen, Germany

^3^Department of Psychiatry, Psychotherapy, and Psychosomatics, Uniklinik RWTH Aachen, Germany
^4^Research Centre Jülich, Institute of Neuroscience and Medicine (INM-1), Jülich, Germany
^5^Research Centre Jülich, Institute of Neuroscience and Medicine (INM-4), Jülich, Germany
^6^School for Physiotherapy, Uniklinik RWTH Aachen, Germany
^7^Department of Clinical Neurophysiology, Georg-August-University, Göttingen, Germany

*** Correspondence:** Prof. Ferdinand Binkofski, Institute of Neuroscience and Medicine (INM-4), Research Centre Jülich, Wilhelm-Johnen-Straße, 52425 Jülich, Germany.
f.binkofski@fz-juelich.de

1. **Supplementary Tables**

**Table S1. Results of the patients in the AAT: language modalities.**

**Abbreviations:** T1, T2, pre- and post-test; TT, Token Test; REP, repetition; WRI, written language; NAM, picture naming; COMP, language comprehension; PH, profile height; *, significant improvement; (*), notable improvement; °, significant deterioration; (°), notable deterioration; all based on the psychometric single-case diagnosis (Huber, 1973) with p<0.01.

TT: error scores; all other modalities: raw scores.

| Patient | 1 | | | | 2 | | | | 3 | | | | 4 | | | |
| --- | --- | --- | --- | --- | --- | --- | --- | --- | --- | --- | --- | --- | --- | --- | --- | --- |
|  |  | | | |  | | | |  | | | |  | | | |
|  | T1 | | T2 | | T1 | | T2 | | T1 | | T2 | | T1 | | T2 | |
|  | R | P | R | P | R | P | R | P | R | P | R | P | R | P | R | P |
| COM | 4 |  | 4 |  | 4 |  | 4 |  | 1 |  | 1 |  | 1 |  | 1 |  |
| ART | 3 |  | 4(*) |  | 5 |  | 4(°) |  | 2 |  | 3(*) |  | 3 |  | 3 |  |
| AUT | 5 |  | 4(°) |  | 5 |  | 5 |  | 1 |  | 2(*) |  | 2 |  | 2 |  |
| SEM | 4 |  | 4 |  | 4 |  | 4 |  | 1 |  | 0(°) |  | 0 |  | 0 |  |
| PHO | 4 |  | 4 |  | 5 |  | 5 |  | 0 |  | 0 |  | 0 |  | 0 |  |
| SYN | 4 |  | 4 |  | 5 |  | 5 |  | 0 |  | 0 |  | 0 |  | 0 |  |
|  |  |  |  |  |  |  |  |  |  |  |  |  |  |  |  |  |
| TT | 3 | 95 | 3 | 95 | 1 | 97 | 0 | 99 | 30 | 46 | 22* | 60 | 48 | 07 | 49 | 5 |
| REP | 114 | 54 | 125(*) | 67 | 150 | 99 | 150 | 99 | 41 | 16 | 30(°) | 14 | 79 | 28 | 85 | 32 |
| WRI | 57 | 59 | 68(*) | 70 | 86 | 96 | 85 | 95 | 11 | 22 | 13 | 24 | 1 | 11 | 6 | 18 |
| NAM | 110 | 94 | 107 | 89 | 119 | 100 | 119 | 100 | 4 | 14 | 15(*) | 18 | 32 | 26 | 14° | 18 |
| COMP | 108 | 95 | 104 | 91 | 113 | 99 | 115 | 100 | 62 | 30 | 70 | 38 | 59 | 27 | 77(*) | 47 |
| PH | 57.9 |  | 58.7 |  | 72.5 |  | 73.2 |  | 41.9 |  | 43* |  | 40.9 |  | 41.3 |  |

**Table S2. Results of the patients in the LEMO-subtests.
Abbreviations:** T1, T2, pre- and post-test; P, performance; LD, Lexical Decision; FS, Finding Synonyms; FR, Finding Rhyms; ON, Oral Naming; -, not feasible; g, guessing performance area; i, impaired performance area; n, normal performance area; *, significant improvement (McNemar Test, p<0.05; (*); °, significant deterioration (McNemar Test, p<0.05).

| Pat. | 1 | | | | 2 | | | | 3 | | | | | 4 | | | | |
| --- | --- | --- | --- | --- | --- | --- | --- | --- | --- | --- | --- | --- | --- | --- | --- | --- | --- | --- |
|  | T1 | P | T2 | P | T1 | P | T2 | P | T1 | P | | T2 | P | T1 | | P | T2 | P |
| LD | 78 | n | 79 | n | 80 | n | 80 | n | 45 | g | | 61* | i | 70 | | i | 74 | n |
| FS | 38 | n | 39 | n | 40 | n | 40 | n | 34 | i | | 36 | i | 35 | | i | 37 | n |
| FR | 11 | g | 7° | g | 20 | n | 20 | n | 10 | g | 6 | | g | | - | - | - | - |
| ON | 19 | n | 20 | n | 18 | i | 18 | i | - | - | | - | - | 9 | | i | 9 | i |

**Table S3. Results of the patients in the AMDNS (degree of impairment).**Degree of impairment: 0 = no impairment; 3 = severe impairment.
**Abbreviations:** T1, T1; Pre- and post-test; DIA, diadochokinesis; DU, duration of phonation; INT, 'variability of speech intensity. (*), notable improvement (change of degree of impairment), (°), notable deterioration (change of degree of impairment).

| Patient | | 1 | | 2 | | 3 | | 4 | |
| --- | --- | --- | --- | --- | --- | --- | --- | --- | --- |
|  | | T1 | T2 | T1 | T2 | T1 | T2 | T1 | T2 |
| DIA | /pa/ | 3 | 1(*) | 3 | 3 | 3 | 3 | 3 | 3 |
|  | /ta/ | 3 | 2(*) | 3 | 3 | 3 | 3 | 3 | 3 |
|  | /ka/ | 3 | 3 | 3 | 3 | - | - | 3 | 3 |
|  | /pataka/ | 3 | 3 | 3 | 3 | - | - | - | - |
|  | /mimamu/ | 3 | 3 | 3 | 3 | - | - | - | - |
|  | /lalilu/ | 3 | 3 | 3 | 3 | - | - | - | - |
|  |  |  |  |  |  |  |  |  |  |
| DU | /sch/ | 1 | 2(°) | 0 | 1(°) | - | - | 3 | 3 |
|  | /s/ | 1 | 2(°) | 0 | 0 | - | - | 3 | 3 |
|  | /f/ | 1 | 0(*) | 2 | 0(*) | - | - | 3 | 3 |
|  | /a/ | 2 | 0(*) | 0 | 0 | 2 | 1(*) | 2 | 3(°) |
|  | /i/ | 2 | 0(*) | 0 | 1(°) | 1 | 2(°) | 3 | 3 |
|  | /u/ | 2 | 1(*) | 1 | 1 | 3 | 3 | 2 | 2 |
| INT |  | 0 | 3(°) | 3 | 0(*) | 0 | 2(°) | 3 | 3 |
|  |  |  | |  | |  | |  |  |
